# Effects of Wet-Pressing and Cross-Linking on the Tensile Properties of Carbon Nanotube Fibers

**DOI:** 10.3390/ma11112170

**Published:** 2018-11-02

**Authors:** Hyunjung Cho, Jinwoo Lee, Haemin Lee, Sung-Hyun Lee, Junbeom Park, Cheol-Hun Lee, Kun-Hong Lee

**Affiliations:** 1 Department of Chemical Engineering, Pohang University of Science and Technology, Pohang 37673, Korea; silver4@postech.ac.kr (H.C.); mcl3395@postech.ac.kr (J.L.); balla1957@postech.ac.kr (H.L.); lch847@postech.ac.kr (C.-H.L.); 2 Advanced Composite Materials, Korea Institute of Science and Technology, Jeonbuk 55324, Korea; sunghyun.lee@kist.re.kr (S.-H.L.); junbeom.park@kist.re.kr (J.P.)

**Keywords:** densification, capillary pressure, cross-linking reaction, specific strength

## Abstract

To increase the strength of carbon nanotube (CNT) fibers (CNTFs), the mean size of voids between bundles of CNTs was reduced by wet-pressing, and the CNTs were cross-linked. Separate and simultaneous physical (roller pressing) and chemical methods (cross-linking) were tested to confirm each method’s effects on the CNTF strength. By reducing the fraction of pores, roller pressing decreased the cross-sectional area from 160 μm^2^ to 66 μm^2^ and increased the average load-at-break from 2.83 ± 0.25 cN to 4.41 ± 0.16 cN. Simultaneous injection of crosslinker and roller pressing augmented the cross-linking effect by increasing the infiltration of the crosslinker solution into the CNTF, so the specific strength increased from 0.40 ± 0.05 N/tex to 0.67 ± 0.04 N/tex. To increase the strength by cross-linking, it was necessary that the size of the pores inside the CNTF were reduced, and the infiltration of the solution was increased. These results suggest that combined physical and chemical treatment is effective to increase the strength of CNTFs.

## 1. Introduction

Carbon nanotube fibers (CNTFs) can be used as 1-dimensional reinforcements of composite materials and could be the next generation of carbon fibers (CFs), which are already used as commercialized reinforcements [1]. CNTFs have several advantages over CF. First, CNTFs have extraordinarily strong (~100 GPa, [2]) building blocks (CNTs). Second, CNTFs are cheaper to synthesize because the synthesis temperature and time of direct spun CNTF (~1000 °C, s, [3]) are lower and shorter than those of CF (>1500 °C, h, [4]); and CNTFs do not require an oxidative stabilization step. Lastly, CNTF is more flexible than CF judging by the high knot efficiency of 100% than that of CF [5,6]. Therefore, the development of strong CNTF would replace the use of CF to CNTF for composite materials.

If the strength of CNTs can be achieved in CNTFs, i.e., if CNTFs that have ~90% of the strength of CNTs can be achieved [7], CNTFs would be a revolutionary addition to the palette of light and strong materials, and would have wide applications in aerospace, architecture, automotive manufacturing, and the military [8]. Currently, the strength of CNTFs (~3.5 GPa [9]) is far less than that of individual CNTs (~100 GPa [2]), so various methods to strengthen CNTFs have been studied.

Post-treatment methods to improve the strength of CNTFs can be divided into physical approaches and chemical approaches. Physical approaches reduce the volume of voids, increase the alignment of CNTs [10,11], and aim to increase the van der Waals (vdW) force (2–10 kJ/mol) between adjacent CNTs. This strategy is used because voids reduce load transfer and weaken CNTFs. Physical approaches that have been tested include torsion [12,13,14], tension [15,16,17,18], and capillary densification [12,14]. Chemical approaches exploit strong covalent bonds (125–500 kJ/mol) between the CNTs [19]. Methods include cross-linking using diazonium salt [20,21,22], radical reactions [23,24,25], [2+1] cycloaddition [26], esterification [27], and plasma treatment [28].

The covalent bonds between CNTs (cross-linked CNTs) strengthened CNTFs, in addition to the vdW force. In practice, the maximization of the strength of CNTs requires a combination of both the physical removal of pores and chemical cross-linking. However, the removal of voids impedes subsequent infiltration of crosslinker chemicals into the CNT bundle, and the chemical reaction impedes the subsequent physical removal of voids because fibers become brittle after cross-linking.

**Figure 1:**
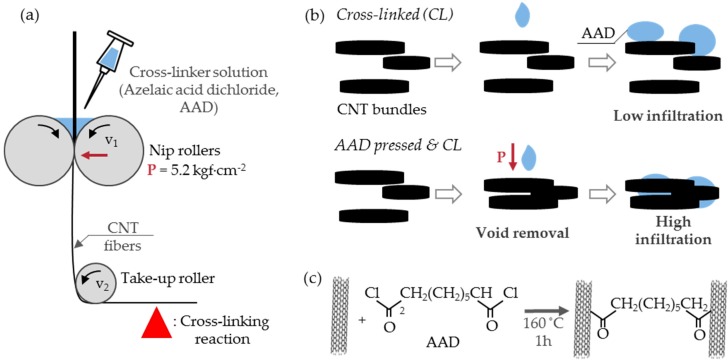
The schematic diagrams of (**a**) experimental procedure, (**b**) effect of roller pressing on solvent infiltration: (up) no pressing, and (down) while pressing; and (**c**) cross-linking reaction.

Here, we propose a strategy to increase the strength of CNTFs by removing pores and cross-linking CNTs simultaneously. CNTFs were roller pressed while the crosslinker solution was injected (wet-pressing) and the Friedel–Craft acylation reaction was used to cross-link the CNTs (Figure 1a–c). Roller pressure and capillary pressure effectively reduced the porosity and increased the infiltration of the crosslinker solution into CNTFs. This approach enabled the identification of the effects of each treatment (i.e., roller pressing, capillary pressure, cross-linking) and increased the strength of CNTFs.

## 2. Materials and Methods

### 2.1. Materials

CNTFs were synthesized by the chemical vapor reaction [29] and were composed of single-walled carbon nanotubes (SWCNTs). The average diameter of SWCNT was 1.2 nm (n = 30) (Figure S1a,b). Thermogravimetric analysis (TGA) results (Figure S1c) showed that CNTFs contained an amorphous carbon impurity and absorbed water of 20.6 wt% and a catalytic impurity of 21.2 wt%. The I_G_/I_D_ ratio was 7.9 ± 0.3 (n = 4) judging by the Raman spectra (Figure S1d). The tubes were compressed using a custom-made roller machine (Figure 1a and Figure S2) composed of nip rollers (diameter = 9.2 cm) and a take-up roller (diameter = 4 cm). The straight distance between the center of the nip rollers and the surface of the take-up roller was 0.3 m. Rollers were made of Teflon, and the nip rollers were wrapped in Teflon tape (width: 10 cm) to reduce damage to the CNTFs while pressing.

### 2.2. Methods

Pristine CNTFs were compared with CNTFs that had been treated in one of four ways (Table 1). (1) Simple cross-linking (*CL*): CNTFs were merely immersed in crosslinker solution (azelaic acid dichloride (AAD), 98%, Aldrich) for 10 min and were taken out, then heated in an Ar atmosphere for 1 h at 100 °C (*CL100*), 130 °C (*CL130*), or 160 °C (*CL160*). After the reaction, samples for Fourier transform infrared (FT-IR) and x-ray photoelectron spectroscopy (XPS) were washed several times with acetone during filtration and dried in an oven at 60 °C. (2) *Pressed* fibers: CNTFs were passed between nip rollers under an applied load of 510 kPa. The velocities of the nip rollers were ~2 m/min and the take-up roller always rotated ~3 mm/min faster than the nip rollers. (3) *Acetone Pressed*: CNTFs were passed through the nip rollers while acetone (99.5%, Sigma-Aldrich) was dropped at 0.6 mL/min on the top of them. The acetone-containing sample was then dried at room temperature for 1 h. (4) *AAD pressed and CL*: CNTFs were passed through the nip rollers while AAD crosslinker was dropped on them at 0.6 mL/min, then the fibers were heated in an Ar atmosphere for 1 h at 160 °C to induce the covalent cross-linking between CNTs.

### 2.3. Characterization

The morphology of CNTFs was determined using field emission scanning electron microscopy (FE-SEM, Philips, XL30 FEG, Amsterdam, The Netherlands). The cross-section was observed using a focused ion beam (FIB, Helios-Pegasus, FEI company, Hillsboro, OR, USA), SEM, and transmission electron microscopy (TEM, JEM-2200FS, JEOL, Tokyo, Japan) [18]. The length or the cross-sectional area *A* (μm^2^) in the SEM images were measured using the ImageJ program (LOCI, University of Wisconsin, Madison, WI, USA). The contact angle of the liquid and the CNT film were measured using SmartDrop (SmartDrop Lab, Seongnam, Gyeonggi, Korea). CNT film (width: 9 cm, height: 4 cm) for the measurement of the contact angle was obtained by repeatedly collecting the CNTFs on a bobbin made of Teflon, then pressing them using the nip rollers while acetone was injected. The results of the chemical reactions were confirmed using FT-IR (Varian, 670/620, Varian, Palo Alto, CA, USA), and XPS (ESCALAB 250, VG Scientific, East Grinstead, Sussex, UK). TGA (STA7300, Hitachi, Tokyo, Japan) was operated at air (heating rate: 5 °C/min). Raman spectra (Horiba Jobin-Yvon LabRam Aramis spectrometer, Edison, NJ, USA) were recorded at the 514 nm line of the Ar-ion laser as the excitation source. The load-at-break *L_B_* (cN) of CNTFs was measured using a FAVIMAT single-fiber tester (FAVIMAT-AIROBOT2, Textechno, Mönchengladbach, NRW, Germany) at an elongation rate of 2 mm·min^−1^ (the number of measurements per sample n = 6–8) and a gauge length of 20 mm. The linear density (tex) of CNTFs were measured by the vibroscopic method using the FAVIMAT, and the specific strength *SS* (N/tex) was calculated [29]. Tensile strength *TS* (GPa) was calculated as *L_B_*/*A*.

## 3. Results and Discussions

### 3.1. Physical Effects of Roller Pressing and Liquid Injection

**Figure 2:**
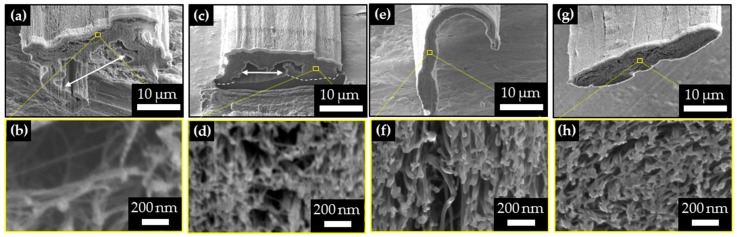
The cross-section of CNTFs: (**a**,**b**) pristine, (**c**,**d**) *CL160,* (**e**,**f**) *pressed*, (**g**,**h**) *AAD pressed and cross-linked*. Pressing eliminated several micrometer-size voids and AAD injection while pressing aggregated bundles. White arrows in (**a**,**b**) indicate the micrometer-size voids > 10 μm.

We observed the cross-section of CNTFs and investigated the physical effects of roller pressing and the injected liquid (Figure 2a–h). Pristine CNTF produced by the direct spinning method had irregular cross-sectional shapes and had macroscopic voids, including one >10 μm (Figure 2a,b, white arrows); they occur when CNTFs are synthesized using direct spinning. CNT agglomeration is produced only at a certain radius of the reactor and hence forms CNT sock having a doughnut-shape in cross-section [30]. The voids reduce the load transfer and *L_B_* of the fibers; moreover, *A* increases as the total volume of the pores increases, so *TS* decreases. Therefore, direct spun CNTFs require post-treatment to reduce porosity and increase strength.

**Figure 3:**
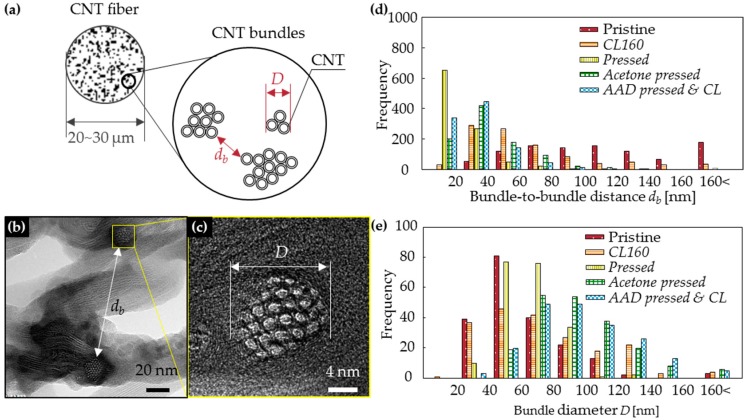
(**a**) The schematic diagram and (**b**,**c**) TEM images of the hierarchical structure of CNTF. The effects of pressing on the distribution of the structures of CNTF: (**d**) bundle-to-bundle distances *d_b_* of CNTF and (**e**) bundle diameters *D*.

The first purpose of roller pressing is to reduce the voids between the bundles. Roller pressing decreased the number of micrometer-size pores, *A*, and bundle-to-bundle distance *d_b_* (nm) (Figure 2a–h and Figure 3d). Compaction eliminated several micrometer-size voids (Figure 2e,g) compared to the uncompacted samples (Figure 2a,c). As a result, *A* of CNTF decreased significantly after roller pressing. Pristine samples had *A* = 160 μm^2^, *CL160* samples had *A* = 110 μm^2^, and the *Pressed* sample had *A* = 66 μm^2^ (41% of *A* in pristine samples). *Acetone pressed* samples had *A* = 85 μm^2^ and *AAD pressed and CL* samples had *A* = 96 μm^2^ which were slightly increased compared to the *pressed* sample. Judging by the shape change of the cross-section from flat (Figure 2e) to oval (Figure 2g), the increase of *A* is presumed to be caused by the surface tension of AAD (36.5 mN·m^−1^), which caused the rearrangement of CNTs inside the CNTFs after passing the pressing rollers with the solution.

Physical compression reduced *d_b_* from hundreds of nanometers to tens of nanometers. The *d_b_* of CNTFs (schematic: Figure 3a; TEM images: Figure 3b) were measured (n = 1000) (Figure 3d). In pristine CNTFs, the distribution of *d_b_* was wide (range, 20 nm to 160 nm). In the *CL160* samples, the most frequent range of *d_b_* was 20–40 nm, but the high end of *d_b_* (of *CL160*) still reached 140 nm; i.e., the porosity was lower than in the pristine samples, but the pore distribution remained wide. On the other hand, *pressed* fibers showed a narrow *d_b_* distribution, mostly <40 nm. Moreover, both the median (179 to 65 nm) and mean values (206 to 71 nm) decreased to about one third after pressing. When the solution was dropped on the samples during pressing, the overall distribution of *d_b_* increased slightly compared with the case where the solution was not used, probably due to the surface tension of the liquid caused aforementioned rearrangement, but most *d_b_* were <40 nm.

The second purpose of roller pressing was to increase the infiltration of the liquid (acetone or AAD). In this experiment, the liquid (AAD) had two functions: as a capillary-pressure inducer and as a crosslinker. CNTFs can be strengthened by capillary densification [12,14] or cross-linking methods [15,16,17,18,19,20,21,22,23]. However, the liquid cannot smoothly infiltrate the CNTF [18] (Figure 1b top), because CNTs are generally insoluble and difficult to disperse [31]. The process of roller pressing while injecting the liquid was designed to increase the infiltration of the solution. Pore reduction and the infiltration of the liquid were facilitated by the external force of the nip rollers (Figure 1b bottom); these changes would increase the effects of AAD, i.e., the CNT bundle aggregation by capillary pressure and cross-linking.

**Figure 4:**
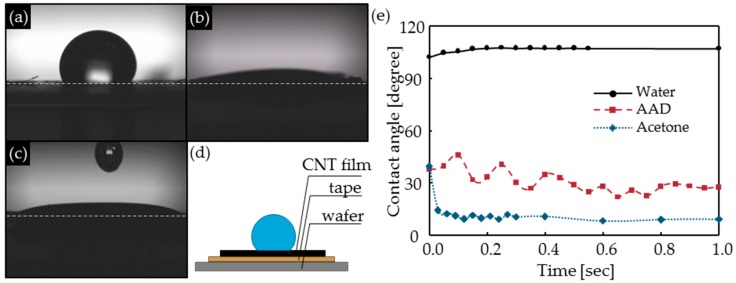
The contact angle to the CNT films of liquid: (**a**) water, (**b**) acetone, and (**c**) azelaic acid dichloride (AAD). (**d**) Schematic diagram of contact angle measurements method. (**e**) The contact angle of liquids vs. time. Slow-motion videos available (Supporting materials).

If the volume and surface area of the pores in CNTFs are constant, the driving force of capillary pressure is *γ*_LV_*·*cos*θ*, where *γ*_LV_ (mN·m^−1^) is the surface tension of the liquid on the liquid/vapor surface, *θ* (°) is the contact angle of the liquid and CNTFs [18]. Here, we measured the contact angle of the CNT films, which provided easier measuring environments than CNTFs. The measured *θ* of acetone (9°) and AAD (27.3°) were much lower than water (106.7°) (Table 2, Figure 4a–e). *γ*_LV_*·*cos*θ* of AAD (32.8 mN·m^−1^) was slightly higher than acetone (22.6 mN·m^−1^), but both were much higher than that of water (−20.7 mN·m^−1^). Therefore, acetone and AAD are better than water to aggregate CNT bundles by capillary pressure.

Diameters *D* (nm) of bundles (Figure 2a–h and Figure 3a,c) were measured (n = 200) and their distributions (Figure 3e) showed the degree of bundle aggregation by capillary pressure. Pristine CNTFs usually had 20 ≤ *D* ≤ 80 nm (average = 61 nm); after roller pressing, most bundles had 40 ≤ *D* ≤ 80 nm (average = 64 nm). This result shows that simple roller pressing did not increase the bundle aggregation significantly, although the process decreased the void sizes to tens of nanometers.

In *acetone-pressed* and *AAD pressed and CL* specimens, most of the bundles had *D* > 60 nm (average ~94 nm) (Figure 3e). This increase compared to roller-pressed CNTFs may be a result of capillary pressure and roller pressing. Capillary pressure is a microscopic phenomenon that occurs between CNTs or between CNT bundles and is considered to be effective for bundle aggregation [14,18], whereas roller pressing is a macroscopic phenomenon that effectively reduces the distances between bundles.

Simple immersion of the CNTF in liquid could not effectively induce capillary pressure. In *CL160* specimens, which were simply dipped in AAD and cross-linked, *D* was almost identical to that of pristine CNTFs, i.e., bundles in *CL160* did not aggregate, as bundles in *acetone pressed* or *AAD pressed and CL* did. This failure may be due to the insufficient infiltration of AAD molecules into the fibers. This result confirms that the concurrent roller pressing and dropping of liquid improved its infiltration effectively.

To summarize, external pressure by roller pressing effectively reduced the voids by several micrometers in size; and internal pressure by the capillary effect caused the aggregation of bundles. Therefore, simultaneous roller pressing and crosslinker liquid infiltration could (i) reduce the void fraction, (ii) aggregate bundles, and (iii) increase the infiltration of the crosslinker.

### 3.2. Chemical Cross-Linking

The vdW force (2–10 kJ/mol) acts between CNTs, but is relatively weak compared to covalent bonds (150–500 kJ/mol) [19]. Therefore, a method to introduce covalent bonds between the CNTs was attempted using the 1-step Friedel-Craft reaction (Figure 1c) with AAD. Among the chemicals having acyl-chloride functional groups at both ends of the chain, AAD was chosen for the following criteria: whether it is liquid at room temperature (for easier handling than vapor and better infiltration than solid), the length of the chain, and the price of the chemicals. The reaction was confirmed by FT-IR spectroscopy and XPS.

**Figure 5:**
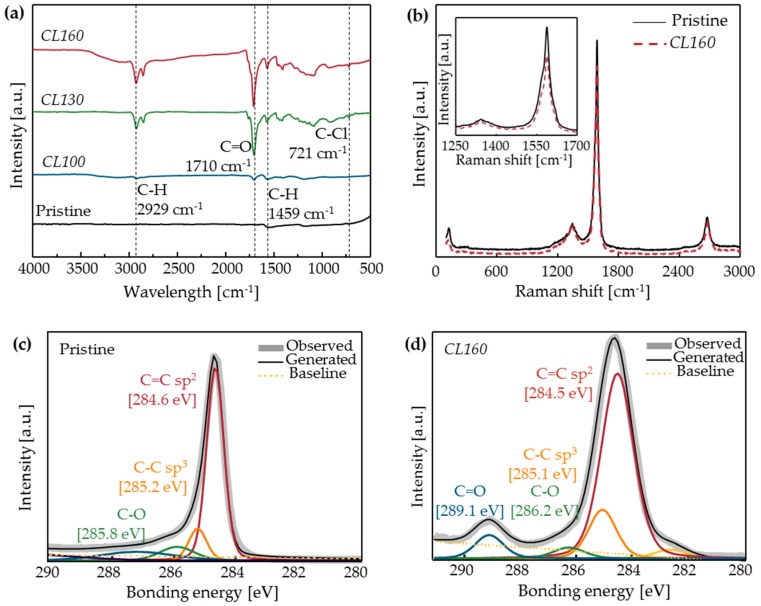
(**a**) The FT-IR spectroscopy of CNTs after cross-linking reactions at various temperatures. (**b**) Representative Raman spectra of the pristine CNTF and *CL160*. XPS of (**c**) pristine CNTs and (**d**) *CL160*. Cross-linking reaction was successful at 160 °C.

The cross-linking reaction was successfully performed at temperatures ≥130 °C. In the pristine sample and *CL100* samples, the FT-IR and XP spectra showed no peaks. In the *CL130* and *CL160* samples, a ketone peak (C=O) occurred at 1710 cm^−1^ and the C–Cl peak was barely observed (Figure 5a); this result means that the C–Cl bond broke and a ketone formed on the CNT surface. The peaks with very low intensities at 721 cm^−1^ (C–Cl) would be caused by the one-end reacted crosslinker. Raman spectra results showed that the I_G_/I_D_ ratio decreased by about 10% from 7.9 ± 0.3 (pristine) to 7.1 ± 0.5 (*CL160*) (n = 4) (Figure 5b). The decreased intensity of the G-peak at 1588 cm^−1^ represents the increased defects after the chemical reaction. The increased defects on the surface of CNTs provides indirect evidence that the chemical reaction has occurred. The XPS C1s spectrum results also confirmed the success of the reaction at 160 °C (Figure 5c,d). In the pristine sample, the ratio of the sp^2^ carbons, which represent C = C bonds in CNTs, was dominant (Figure 5c). After the chemical reaction, the ratio of sp^2^ carbon decreased and that of sp^3^ carbon increased (Figure 5d). Additionally, a ketone peak at 289.1 eV appeared; which was almost absent in the pristine sample. Lastly, TGA curves also showed the indirect evidence of the successful chemical reaction judging by the decreased thermal stability of CNTs (Figure S3).

### 3.3. Tensile Properties

**Figure 6:**
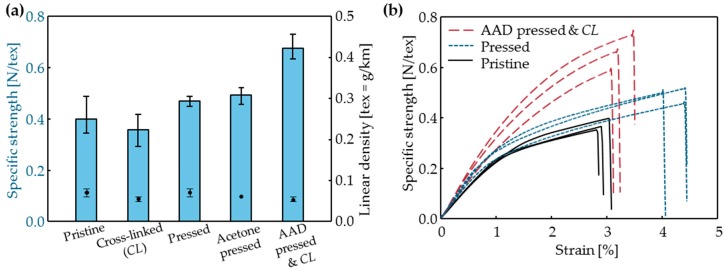
(**a**) The specific strength (bars) and linear density (circles) Error bars: minimum to maximum; and (**b**) Representative stress-strain curve of CNTFs.

Pressing increased *L_B_* of CNTF from 2.83 ± 0.25 cN to 4.41 ± 0.16 cN (Table 3). This change was a result of the elimination of the micrometer-size pores and the reduction in *d_b_*. However, roller compression does not always increase *L_B_*_._ The effect of roller pressing on *L_B_* was affected by the number of pressings, and on the material used to perform them. For example, when the fibers were repetitively pressed, *L_B_* was instead reduced (Figure S4). Additionally, the roller pressing of CNT film between two metal plates decreases *L_B_* but increases *TS* by greatly decreasing *A* [32]. In the present study, the increase of *SS* was small (from 0.40 ± 0.05 N/tex to 0.47 ± 0.01 N/tex after pressing), but the increase in *L_b_* is meaningful (Figure 6a, Table 3).

The combination of physical compression and chemical cross-linking increased the strength of the CNTF. In the *Pressed* sample, the distribution of *SS* narrowed (Figure 6a); i.e., the structure and strength of the CNTF became uniform along its length. Uniformity is important in the fiber because it is directly related to its strength [33]. Therefore, roller pressing has the advantage that it imparts uniformity. The *Acetone pressed* sample had an *SS* = 0.49 ± 0.03 N/tex, which was similar to that of the *pressed* sample (Figure 6a), but the *AAD pressed and CL* sample had *SS* = 0.67 N/tex, which was 67% higher than the *SS* of the pristine sample, 43% higher than the *SS* of the *Pressed* CNTFs, and 37% higher than the *SS* of *Acetone pressed*. The strength of the *CL160* sample, which was cross-linked only without pressing, did not increase compared to that of pristine CNTFs (Figure 6a). This failure of strengthening may be due to the lack of infiltration of the AAD in the *CL160* sample. Therefore, the reduction of the void fraction and increase in the crosslinker molecules’ infiltration are both important factors in the cross-linking method to increase strength.

The stress-strain curve shows the effect of physical and chemical treatments on strain-at-break *S_B_* (%) and toughness (Figure 6b). *S_B_* increased from 3.4 ± 0.5% to 4.3 ± 0.2% after pressing by rollers, then decreased to 3.5 ± 0.4% in the *AAD pressed and CL* sample. Toughness showed a similar trend: it increased from 0.74 ± 0.05 N/tex to 1.43 ± 0.09 N/tex after pressing and became 1.36 ± 0.22 N/tex after AAD pressing and cross-linking. The ductility and the energy absorbed by CNTF before fracture, i.e., toughness, increased after roller pressing; this change may be due to the reduced void fraction and increased shear force. Due to van der Waals forces, CNTFs break as bundles slip and result in the tapered broken end [21]. Therefore, increased van der Waals forces would increase a slip range, which increases the strain-at-break. Generally, after the cross-linking reaction, CNTFs become strong but also become brittle [21,22,25,34,35]. However, in this study, physical properties such as *S_B_* or the toughness of the *AAD pressed and CL* sample were similar or improved compared to the pristine CNTF because chemical treatments were conducted after the structural change by roller pressing.

**Figure 7:**
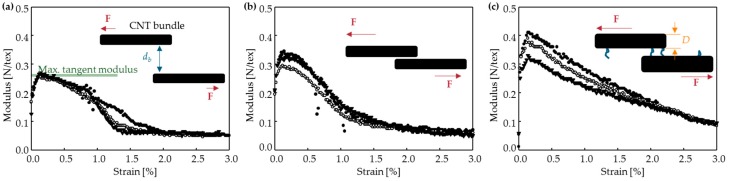
The modulus-strain curves: (**a**) Pristine, (**b**) *pressed*, and (**c**) *AAD pressed and CL*. Maximum modulus increased after the fibers were treated.

The maximum tangent modulus was 0.26 N/tex in the pristine CNTFs, 0.32 N/tex in the *pressed* samples, and 0.37 N/tex in the *AAD pressed and CL* samples. (Figure 7a–c). The slight increase in the maximum tangent modulus after roller pressing may be a result of the increase in shear force as the pore size decreased (Figure 7a,b). In the pristine and *pressed* samples, the modulus remained low (<0.1 N/tex) at a strain of >1.5%, whereas in the *AAD pressed and CL* samples, it was >0.1 N/tex until the strain reached 3%, then it tended to decrease linearly; this result means that the resistance of the CNTF to deformation increased after chemical cross-linking, i.e., a covalent bond formed (Figure 7c). The shape of the modulus-strain curve changes depending on the internal structure of CNTFs [36]; i.e., when the individual CNTs in the CNTF do not act independently but act en masse, the modulus-strain curve changes from a sigmoid decay after the peak at low strain (Figure 7a,b) to a linear decay after the peak (Figure 7c). This change supports the hypothesis that roller pressing reduced the void fraction in the CNTF and that a reaction cross-linked the CNTs.

## 4. Conclusions

The strength of a CNTF can be increased by introducing covalent bonds among CNTs in the CNTF. The prerequisite for this approach, however, is the deep infiltration of crosslinker molecules into the voids in the CNTF. In this paper, we demonstrated that wet-pressing using two rollers is an effective method for this purpose. Deep infiltration was achieved because the average size of the voids is reduced by the physical pressing, resulting in a higher capillary force acting on the narrower gaps. The chance of cross-linking also increases due to the reduction in distance between CNTs and/or CNT bundles. Therefore, wet-pressing and cross-linking, which is the combination of void reduction by the physical method and increase in covalent bonds by the chemical method, effectively increases the strength of CNTFs.

## Figures and Tables

**Table 1:** The sample name and treated methods.

Code	Sample Name	Roller Pressing	Solvent Infiltration	Cross-Linking Reaction (Temperature)	Related Figures
0	*Pristine*	no	no	no	–
1	*CL100*	no	yes (AAD)	yes (100 °C)	Figure 1b (up)
1	*CL130*	no	yes (AAD)	yes (130 °C)	Figure 1b (up)
1	*CL160*	no	yes (AAD)	yes (160 °C)	Figure 1b (up)
2	*Pressed*	yes	no	no	–
3	*Acetone pressed*	yes	yes (Acetone)	no	–
4	*AAD pressed and CL*	yes	yes (AAD)	yes (160 °C)	Figure 1a,b (down)

**Table 2:** The surface tension at 25 °C and 1 atm, and the contact angle to the CNT films of three solvents.

Liquid	Surface Tension $\gamma_{LV}$(mN·m^−1^)	Contact Angle *θ* (°)	$\gamma_{LV}\cdot cos\theta$ (mN·m^−1^)
Water	72.0	106.7	−20.7
Acetone	22.9	9.0	22.6
AAD	36.5	27.3	32.4

**Table 3:** The average ± s.d. (6 ≤ n ≤ 8) mechanical properties of CNTFs.

Factors	Unit	Sample Name				
		Pristine	*CL160*	Pressed	Acetone Pressed	AAD Pressed and C*L*
Specific strength	N/tex	0.40 ± 0.05	0.36 ± 0.04	0.47 ± 0.01	0.49 ±0.03	0.67 ± 0.04
Tensile strength	MPa	180 ± 20	180 ± 10	680 ± 20	350 ± 20	380 ± 20
Load at break	cN	2.83 ± 0.25	1.94 ± 0.15	4.41 ± 0.16	2.98 ± 0.18	3.61 ± 0.22
Linear density	tex	0.07 ± 0.01	0.06 ± 0.00	0.07 ± 0.01	0.06 ± 0.00	0.05 ± 0.00
Cross-sectional area	μm^2^	160	110	66	85	96
Strain at break	%	3.4 ± 0.6	3.6 ± 0.6	4.3 ± 0.2	3.7 ± 0.4	3.5 ± 0.4
Toughness	N/tex	0.74 ± 0.05	0.90 ± 0.29	1.43 ± 0.09	1.31 ± 0.20	1.36 ± 0.22
Maximum tangent modulus	N/tex	0.26 ± 0.01	0.25 ± 0.02	0.32 ±0.02	0.31 ± 0.02	0.37 ± 0.03

## Supplementary Materials

The following are available online at http://www.mdpi.com/1996-1944/11/11/2170/s1, Figure S1: Morphology of pristine CNTF, Figure S2: Custom-made roller machine, Figure S3: TGA curve of pristine CNTs (purchased) and *CL160*, Figure S4: Load-at-break vs. the number of pressing times, the contact angle of CNT film and GIF S1: water, GIF S2: acetone, GIF S3: AAD.
