# Supplementary material for: Effects of Wet-Pressing and Cross-Linking on the Tensile Properties of Carbon Nanotube Fibers
Source: Materials (Basel). 2018 Nov 2;11(11):2170. doi: 10.3390/ma11112170 (PMC6266003; doi:10.3390/ma11112170)
Supplement: Supplementary file 1 [file materials-11-02170-s001.zip › materials-supplementary.pdf]

materials

MDPI

Article-Supplementary Materials

Effects of Wet-Pressing and Cross-Linking on the Tensile Properties of Carbon Nanotube Fibers

Hyunjung Cho [1], Jinwoo Lee [1], Haemin Lee [1], Sung-Hyun Lee [2], Junbeom Park [2], Cheol-Hun Lee [1] and Kun-Hong Lee [1,*]

[1] Department of Chemical Engineering, Pohang University of Science and Technology, Pohang, 37673, Korea; silver4@postech.ac.kr (H.C.); mcl3395@postech.ac.kr (J.L.); balla1957@postech.ac.kr (H.L.); lch847@postech.ac.kr (C.-H.L.)

[2] Advanced Composite Materials, Korea Institute of Science and Technology, Jeonbuk, 55324, Korea; sunghyun.lee@kist.re.kr (S.-H.L.); junbeom.park@kist.re.kr (J.P.)

* Correspondence: ce20047@postech.ac.kr; Tel.: +82-54-279-2271

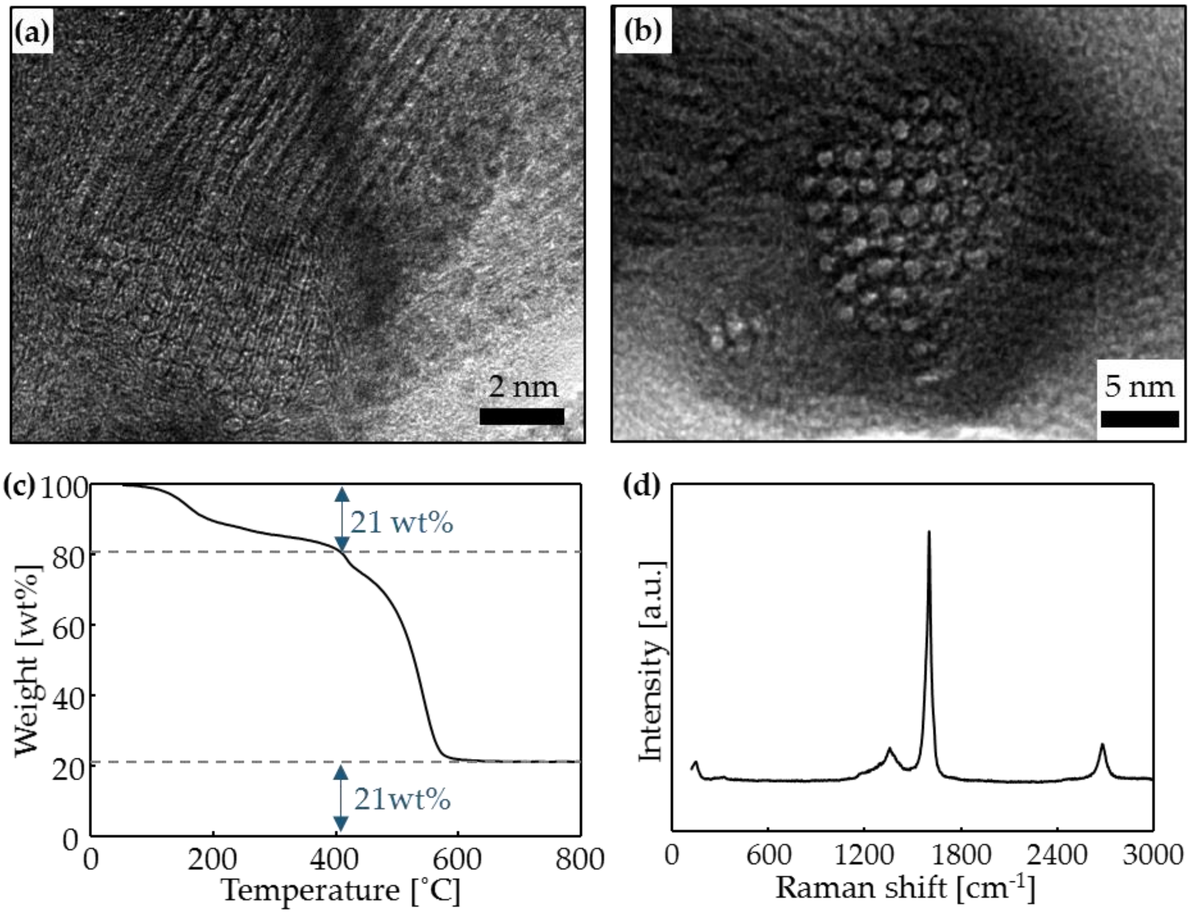

Figure S1. (**a**), (**b**) TEM images, (**c**) TGA curves at air and (**d**) Raman spectra of pristine CNT fiber.

A custom-made roller machine was used to uniformly press CNTFs at constant pressure of 5.2 kgf·cm^{-2} (Figure S2). CNTFs were pressed by passing through the nip rollers and then wound at a take up roller. Liquids were injected at the top of nip rollers.

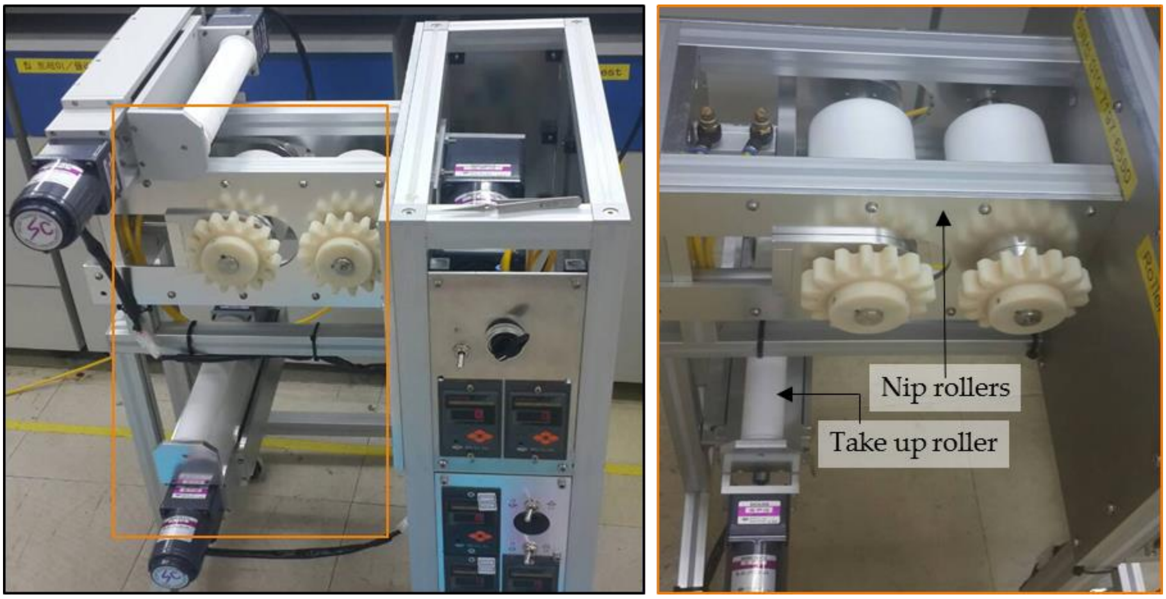

Figure S2. The custom-made roller machine to press CNTFs.

TGA results under air showed the decreased thermal stability of CNTs (Nanostructred & Amorphous Materials Inc. MWCNTs 95%, OD: 10-20 nm, L: 30–100 μm) after cross-linking reaction (Figure S3) due to the effects of the injected cross-linker solution (AAD). The 13 wt% of *CL160*, which is a cross-linker solution (AAD) that has not succeeded in cross-linking reaction, decomposed at less than 276 °C. The decomposition temperature of CNTs in *CL160* was lower than that of CNTs in pristine of 617 °C. Substances that decomposed between 276 °C and 496 °C (about 28 wt% of *CL160*) were thought to be successfully cross-linked cross-linker and CNTs with a high proportion of defects (i.e., sp^3 carbons). Lastly, 59 wt% of *CL160* decomposed at 606 °C seems CNTs with relatively low defects.

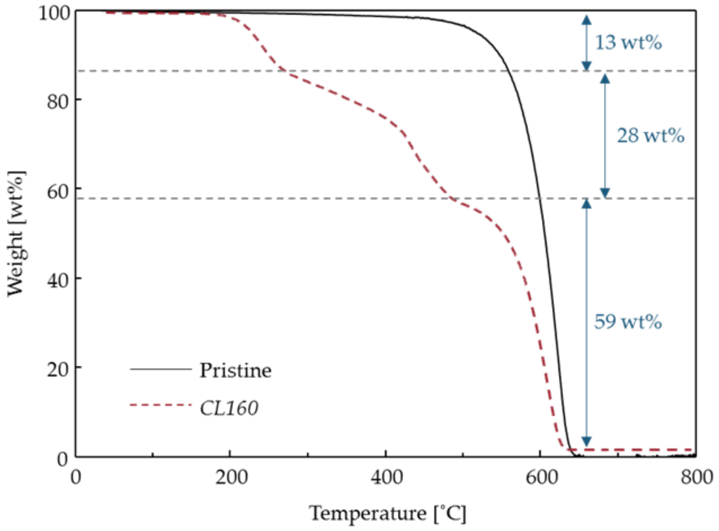

Figure S3. TGA data of the pristine and cross-linked (*CL160*) CNTs (Heating rate: 5 °C/min, at air).

Repeated roller-press was performed to investigate the strength change with the number of pressing times. For roller *pressing & CL samples*, the cross-linker solution was injected only when the final roller pressing was performed. Load-at-failure increased as the number of compactions increased to three, but then decreased (Figure S4). The curvilinear relationship explains the two effects of press on strength. First, the increase occurs because of the reduced size and fraction of voids;

and increased the van der Walls forces between the CNTs. Second, the later decrease occurred because too much compaction damaged the CNTF and lead to decreased load at failure. This shows that optimum degree of pressing is required to practically increase the strength of CNTF, not by the increase in tensile strength due to a reduction in cross-sectional area.

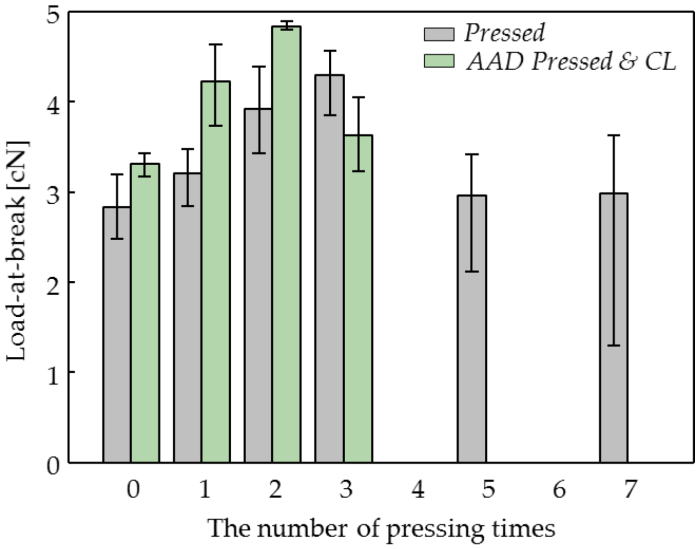

Figure S4. Load-at-break of CNTFs vs. the number of pressing times. The strength increased by the effect of increased load-at-break, not by the effect of decreased cross-sectional area.
