# Supplementary figures and images for: Effects of Wet-Pressing and Cross-Linking on the Tensile Properties of Carbon Nanotube Fibers
Source: Materials (Basel). 2018 Nov 2;11(11):2170. doi: 10.3390/ma11112170 (PMC6266003; doi:10.3390/ma11112170)

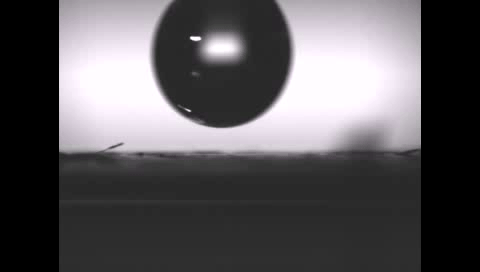

Supplement: Supplementary file 1 [file materials-11-02170-s001.zip › GIF1.contact angle_water.gif]

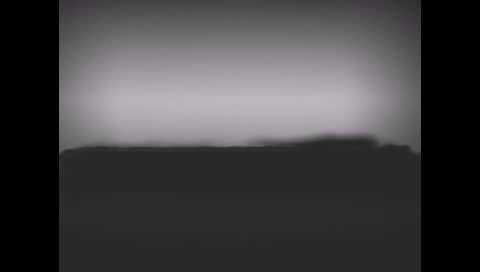

Supplement: Supplementary file 1 [file materials-11-02170-s001.zip › GIF2.contact angle_acetone.gif]

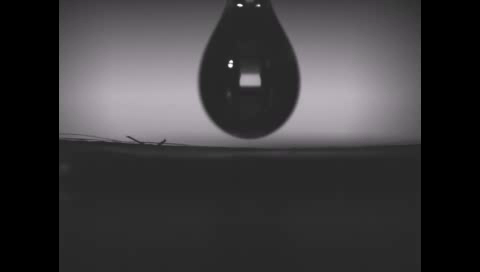

Supplement: Supplementary file 1 [file materials-11-02170-s001.zip › GIF3.contact angle_AAD.gif]
